# γδT Cells Suppress Liver Fibrosis via Strong Cytolysis and Enhanced NK Cell-Mediated Cytotoxicity Against Hepatic Stellate Cells

**DOI:** 10.3389/fimmu.2019.00477

**Published:** 2019-03-15

**Authors:** Meifang Liu, Yuan Hu, Yi Yuan, Zhigang Tian, Cai Zhang

**Affiliations:** ^1^School of Pharmaceutical Sciences, Institute of Immunopharmacology and Immunotherapy, Shandong University, Jinan, China; ^2^School of Life Sciences, Institute of Immunology, University of Science and Technology of China, Hefei, China

**Keywords:** fibrosis, γδT cell, NKp46, cytotoxicity, γδT1, γδT17, CD137, cell crosstalk

## Abstract

Liver fibrosis is the excessive accumulation of extracellular matrix proteins, resulting from maladaptive wound healing responses to chronic liver injury. γδT cells are important in chronic liver injury pathogenesis and subsequent liver fibrosis; however, their role and underlying mechanisms are not fully understood. The present study aims to assess whether γδT cells contribute to liver fibrosis regression. Using a carbon tetrachloride (CCl_4_)-induced murine model of liver fibrosis in wild-type (WT) and γδT cell deficient (TCRδ^−/−^) mice, we demonstrated that γδT cells protected against liver fibrosis and exhibited strong cytotoxicity against activated hepatic stellate cells (HSCs). Further study show that chronic liver inflammation promoted hepatic γδT cells to express NKp46, which contribute to the direct killing of activated HSCs by γδT cells. Moreover, we identified that an IFNγ-producing γδT cell subset (γδT1) cells exhibited stronger cytotoxicity against activated HSCs than the IL-17-producing subset (γδT17) cells upon chronic liver injury. In addition, γδT cells promoted the anti-fibrotic ability of conventional natural killer (cNK) cells and liver-resident NK (lrNK) cells by enhancing their cytotoxicity against activated HSCs. The cell crosstalk between γδT and NK cells was shown to depend partly on co-stimulatory receptor 4-1BB (CD137) engagement. In conclusion, our data confirmed the protective effects of γδT cells, especially the γδT1 subset, by directly killing activated HSCs and increasing NK cell-mediated cytotoxicity against activated HSCs in CCl_4_-induced liver fibrosis, which suggest valuable therapeutic targets to treat liver fibrosis.

## Introduction

Liver fibrosis is a prevalent liver disease that can lead to cirrhosis and liver failure, accounting for about 45% of deaths in industrialized countries (1). Liver fibrosis is characterized by excessive accumulation of scar tissue resulting from ongoing inflammation and liver cell death during chronic liver diseases. The scar tissue, comprising collagen-rich extracellular matrix, replaces normal functional units of cells, leading to the progressive loss of organ function and eventual failure (2). Hepatic stellate cells (HSCs) are the main effector cells in liver fibrosis because of their capacity to transdifferentiate into collagen-producing myofibroblasts (3). In a normal liver, HSCs are quiescent, vitamin A-storing cells that show autofluorescence. Following liver injury, HSCs become activated, lose their vitamin A droplets, and transdifferentiate into extracellular matrix-producing myofibroblasts, which promote hepatic fibrosis (4, 5). Their location in the narrow space of Dissé between hepatocytes and sinusoidal endothelial cells mean that HSCs contact closely with other liver cell types. HSCs sense the changes of tissue integrity and initiate innate immune cell activation. Accordingly, certain immune cells interact with HSCs via cell-cell interactions or by secreting soluble mediators, possibly influencing the progress of liver fibrosis (3).

γδT cells are enriched in the liver, representing 3–5% of total liver lymphocytes, and play an important role in various liver diseases (6, 7). γδT cells are involved in liver fibrosis and appear to show a protective function by directly killing activated HSCs in murine liver fibrosis (8). However, the cellular and molecular mechanism by which γδT cells alleviate liver fibrosis is poorly understood.

Besides their direct role in killing activated HSCs, γδT cells are indispensable to regulate natural killer (NK) cell-mediated antitumor responses in a co-stimulatory receptor 4-1BB (CD137)-dependent manner, suggesting cell-crosstalk between γδT cells and NK cells (9, 10). NK cells also play an important role in alleviating the development of fibrosis by killing activated HSCs (11, 12). Therefore, we sought to determine whether γδT cells could regulate NK cell-mediated cytotoxicity against HSCs through CD137 engagement during the development of liver fibrosis. Recently, hepatic NK cells were subdivided into CD49a^+^DX5^−^ liver-resident NK (lrNK) cells and CD49a^−^DX5^+^ conventional NK (cNK) cells (13–15). The cNK cells protect against liver fibrosis by killing activated HSCs in a TNF-related apoptosis inducing ligand (TRAIL)-dependent manner; however, the role of lrNK cells, with high TRAIL expression, in fibrosis has not been investigated (11). Whether γδT cells could increase the anti-fibrotic effects of lrNK cells or cNK cells is unknown. Therefore, we focused on the mechanism by which γδT cells and NK cells (especially lrNK cells) kill HSCs and the crosstalk between γδT cells and lrNK/cNK cells during the development of liver fibrosis. To achieve these aims, we used a carbon tetrachloride (CCl_4_)-induced mouse model of liver fibrosis in wild-type (WT) and γδT cell deficient (TCRδ^−/−^) mice.

## Materials and Methods

### Animals and Models of Fibrosis

Pathogen-free male C57BL/6J (5–6 weeks old) mice were purchased from HFK Bioscience Co., Ltd. (Beijing, China). C57BL/6J-derived TCRδ^−/−^ mice (TCRδ^−/−^, male, 5–6 weeks old) and C57BL/6J-derived-NKp46^−/−^ (NCR1^gfp/gfp^) mice were purchased from The Jackson Laboratory (Bar Harbor, ME, USA). The genotype identification of TCRδ^−/−^ and NKp46^−/−^ mice were shown in Supporting Figures 1A,B. Experiments were performed with age- and sex-matched animals at 6–12 weeks of age under ethical conditions, according to the guidelines for experimental animals from Shandong University and were approved by the Committee on the Ethics of Animal Experiments of Shandong University. Liver fibrosis was induced by intraperitoneal injections of CCl_4_ (Fuyu, Tianjin, China) at 0.6 mL/kg body weight mixed with corn oil (Sigma-Aldrich Co., St. Louis, MO, USA), thrice-weekly for 4 weeks (8). For the cytokine neutralization experiments, fibrosis-induced mice were simultaneously injected intraperitoneally with 100 μg anti-IL-17A monoclonal antibodie (mAb) (TC11-18H10.1; Biolegend, San Diego, CA, USA) or anti-IFNγ mAb (XMG1.2; Biolegend, San Diego, CA, USA), twice weekly for 4 weeks.

### Histological Analysis and Serum ALT Measurement

Liver tissues were fixed with 4% paraformaldehyde for 24 h, dehydrated, embedded in paraffin and sectioned at 5 μm. The paraffin-embedded sections were stained with hematoxylin and eosin (H&E) according to the standard protocols of our laboratory, as previously described (16). Sirius Red (SR) staining was performed on liver tissue sections to determine liver fibrosis according to a standard protocol (17). In brief, liver sections were stained with 0.4% SR in saturated picric acid for 0.5 h, and then washed, dehydrated and examined by light microscopy. Sirius red-positive areas were quantified in five non-overlapping random fields on each slide using Image J software (NIH, Bethesda, MD, USA). Serum alanine aminotransferase (ALT) activities were measured using an ALT (GPT) kit (Nanjing, China).

### Immunohistochemistry

Liver paraffin sections (5 μm) were deparaffinized and dehydrated, and microwave antigen retrieval was performed following peroxidase quenching with 3% H_2_O_2_ for 10 min. Subsequently, the sections were blocked with 10% goat serum (C0265, Beyotime, Shanghai, China) for 30 min at room temperature (RT). The primary antibody mouse anti-α-smooth muscle actin (α-SMA) antibody (Ab5694, Abcam, Cambridge, U.K.) was diluted 1/200, incubation overnight at 4°C. A Biotin conjugated goat polyclonal to rabbit IgG (SP-9000, ZSGB-BIO, Beijing, China) was used at dilution at 1/200 as the secondary antibody followed by incubation with horseradish peroxidase (HRP)-Streptavidin for 30 min and then staining was detected with 3,3′-diaminobenzidine tetrahydrochloride (DAB) for 5–10 min. For better documentation, the tissue sections were counterstained with hematoxylin.

### Immunofluorescence

The freshly isolated livers were frozen in optimal cutting temperature (O.C.T.) compound (Sakura Finetek, Torrance, CA, USA, Inc.) and tissue sections were sliced into 5 μm-thickness, stored at −80°C. For α-SMA or desmin immunostaining, sections were thawed to RT, fixed with pre-cold acetone for 10 min, and then rehydrated in phosphate-buffered saline (PBS) for 10 min. Thereafter, the sections were permeabilized with 1% Triton X-100 in PBS for 20 min, blocked with blocking buffer (10% goat serum in PBS) for 30 min at RT, and then incubated with primary anti-α-SMA antibody (Ab5694, Abcam, Cambridge, U.K.) or anti-desmin antibody (ab15200, Abcam, Cambridge, U.K.), overnight at 4°C. The sections were then washed three times with PBS and incubated with a secondary AF594-conjugated Goat anti-rabbit IgG (H&L) antibody (SA00006-4, Proteintech, USA) or AF488-conjugated Goat anti-rabbit IgG (H&L) (A0423, Beyotime, Shanghai, China) for 1 h at RT.

For bi-color immunofluorescence staining of α-SMA and γδTCR (T cell receptor) or γδTCR and NKp46, liver sections were incubated with 5% bovine serum albumin (BSA) for 30 min and followed by incubation with anti-TCR gamma + TCR delta antibody [GL-3] [fluorescein isothocyante (FITC)] (ab118864, Abcam, 1/20) and anti-α-SMA antibody (1/200) or with hamster anti-TCRγ/δ antibodies (14-5711-82, Invitrogen, USA, 1:100) and anti-NCR1 antibodies (ab214468, Abcam, 1/200), overnight at 4 °C. After washing, the sections were incubated with a secondary AF594-conjugated Goat anti-rabbit IgG (H&L) antibody or AF488-conjugated Goat anti-rabbit IgG (H&L) and AF594-conjugated Goat anti-hamster IgG (H&L) (405512, Biolegend, USA) for 1 h at RT. After repeated washes, the sections were mounted with 2-(4-amidinophenyl)-1H-indole-6-carboxamidine (DAPI) (C1002, Beyotime, Shanghai, China, 1/1000) and visualized using a confocal microscope (LSM780, Zeiss, Germany) or scanned using the StrataFAXS Plus system (TissueGnostics GmbH, Vienna, Austria) and analyzed by StrataQuest.

### Isolation of Primary HSCs

Primary HSCs were isolated according to the standard protocols of our and other laboratories, as previously described (18, 19). In brief, mouse livers were perfused *in situ* with perfusion buffer containing 0.075% collagenase type I (1723329, Gibco, Carlsbad, CA, USA) and digested with digestion buffer containing 0.009% collagenase type I and 0.02% DNase I (10104159001, Roche, Indianapolis, IN, USA). The cell suspension was centrifuged at 50 × g for 3 min at RT to remove hepatocytes, and repeated three times. The supernatant was centrifuged at 450 × g for 10 min. The cell pellet was then resuspended in 15% OptiPrep gradient (11145421, AXIS-SHIELD PoC AS, Oslo, Norway) and overlayed 11.5% OptiPrep gradient and 1,640 medium. After centrifuging at 1,400 × g, the cell fraction in 1,640 medium and 11.5% OptiPrep interphase was gently aspirated for HSCs isolation. Cell viability was determined by trypan blue staining. Cell purity was confirmed according to its three major characteristics: Its star-like shape, perinuclear lipid droplets, and vitamin A-specific auto-fluorescence (20).

### Immunocytostaining

Primary HSCs were prepared as described above. After seeding on coverslips for 5 days, the adherent-wall cultured primary HSCs were fixed with 4% paraformaldehyde for 15 min at RT and washed with PBST (0.1% Tween-20 in PBS). They were then permeabilized with ice-cold 1% Triton X-100 in PBS for 10 min and blocked with 10% goat serum for 1 h at RT. The cells then were incubated with primary antibodies which were diluted in PBS with 0.5% BSA for overnight at 4°C. Cells were washed with PBS three times and incubated with diluted fluorochrome-conjugated secondary antibodies for 1 h at RT. DAPI was used for nucleus staining. After repeated washes, the cells were mounted and viewed under a confocal microscope.

Terminal deoxynucleotidyl transferase-mediated dUTP nick-end-labeling (TUNEL) assays were performed using a commercially available kit (C1089, Beyotime, Shanghai, China) following the manufacturer's instructions. Thereafter, the coverslips were incubated with 5% BSA for 30 min at RT and then incubated with anti-α-SMA primary antibodies at 37°C for 1 h. Coverslips were washed with PBST three times and then incubated with the secondary Alexa Fluor 488-labeled Goat Anti-Rabbit IgG (H+L) antibody for 1 h at RT, washed three times, and incubated with DAPI for 5 min. Fluorescent images were visualized using a confocal microscope.

### Flow Cytometry Analysis and Cell Sorting

Liver mononuclear cells (LMNCs) were isolated as previously described (21). In brief, mouse livers were freshly harvested and LMNCs were isolated by density gradient centrifugation. The LMNCs were counted using an automated cell counter (TC20, Bio-Rad, Hercules, CA, USA).

Fluorescent mAbs against, CD49a (Ha31/8), CD49b (DX5), γδTCR (GL3), NKp46 (29A1.4), NKG2D (CX5) were purchased form BD Biosciences (San Jose, CA, USA). mAbs against γδTCR (eBioGL3) and TRAIL (N2B2) were purchased from eBioscience. Abs against CD3e (145-2C11), NK1.1 (Killer cell lectin-like receptor subfamily B, member 1; PK136), NKG2A (Natural Killer Group Protein 2; 16A11), CD69 (H1.2F3), CD137 (17B5), CD107a (1D413), DNAM-1 (DNAX accessory molecule 110E5), interferon gamma (IFNγ) (XMG1.2), IL-17A (Tc11-18H10), Fas ligand (FasL) (MFL3), granzyme B (GmB) (GB11) were purchased from Biolegend. After blocking non-specific Fc receptor (FcR) binding with anti-CD16/32 (eBioscience), the LMNCs were then stained with the indicated fluorescent mAbs for surface molecules. For intracellular markers, cells were stimulated with phorbol myristate acetate (PMA) and ionomycin. After surface staining, cells were fixed, permeabilized, and stained with the mAbs against the indicated intracellular molecules or isotype control Abs. Cells were analyzed by fluorescence activated cell sorting (FACS) Aria III (BD). Data were analyzed using the Flow Jo software (Treestar Inc., Ashland, OR, USA).

### Adoptive Transfer of γδT Cells

The adoptive transfer of γδT cells assay was performed as described previously (8). Liver γδT cells of WT mice were isolated using FACS-sorting. About 100,000 γδT cells or normal saline in the same volume were intravenously injected into TCRδ^−/−^ mice once a week following treatment with CCl_4_ thrice weekly for 4 weeks.

### Co-culture Experiments and Lysis Assay

Primary HSCs, which were isolated by density gradient centrifugation as described above, were cultured in 1,640 medium in 96-well plates at 10,000 cells per well for 5 days, developing culture-activated HSCs. The cytolytic activities of γδT cells, lrNK cells, or cNK cells were assessed using a lactate dehydrogenase (LDH) release assay. The culture-activated HSCs were used as target cells. Liver γδT cells, lrNK cells, and cNK cells were isolated from livers of CCl_4_-treated WT mice. These isolated cells were incubated with culture-activated HSCs at an effector:target ratio of 2:1 in the presence or absence of specific blocking antibody for 12 h, respectively. To detect the cytotoxicity of γδT cells and NK cells after CD137L blockade against activated HSCs, γδT cells isolated from CCl_4_-treated WT mice were co-cultured with lrNK cells or cNK cells isolated from CCl_4_-treated TCRδ^−/−^ mice at the ratio of 1:1. These co-cultured cells were then incubated with activated HSCs at an effector:target ratio of 2:1 in the presence or absence of anti-CD137L blocking antibody (107108, Biolegend, San Diego, CA, USA) for 12 h. Cytotoxicity was measured using an LDH Cytotoxicity Assay Kit (C0017, Beyotime, Shanghai, China). Cytotoxicity was calculated as follows:

$$\begin{array}{l} \text{lysis }(\%)=[1-(\text{A of cocultured cells}-\text{A of effector cells})/\\ \text{ A of target cells}]\times 100\%. \end{array}$$

A, absorbance.

The time-lapse cytotoxicity of γδT cells, lrNK cells, and cNK cells against activated HSCs was measured by counting the numbers of adherent live HSCs at different time points using a Biotek Cytation 5 V. In brief, primary HSCs were isolated and cultured in 24-well plates at 100,000 cells per well for 5 days. γδT cells, lrNK cells, and cNK cells from the livers of CCl_4_-treated WT mice were obtained by FACS-sorting and labeled with carboxyfluorescein succinimidyl ester (CFSE). These CFSE-labeled effector cells were incubated with culture-activated HSCs at an effector:target ratio of 2:1 for 12 h. The numbers of CFSE-negative adherent HSCs were monitored in real time using a Biotek Cytation 5V. Cytotoxicity was calculated as follows:

Lysis (%) = (1- the numbers of CFSE-negative adherent HSCs at indicated time point/the numbers of CFSE-negative adherent HSCs at the initial time after co-culturing) × 100%.

### Statistical Analyses

The data were statistically analyzed by the Statistical Package for the Social Sciences (SPSS) software (V.16.0, SPSS Inc., Chicago, IL, USA). Data are presented as the mean ± standard error of the mean (SEM). Differences among more than two groups were assessed by one-way analysis of variance (One-Way ANOVA) and differences between two groups were assessed using Independent-Samples *T*-Test. A *P*-value < 0.05 was considered statistically significant (^*^P < 0.05; ^**^P < 0.01; ^***^P < 0.001).

## Results

### γδT Cell Deficiency Exacerbates Liver Fibrosis Upon Chronic Liver Injury

**Figure 1:**
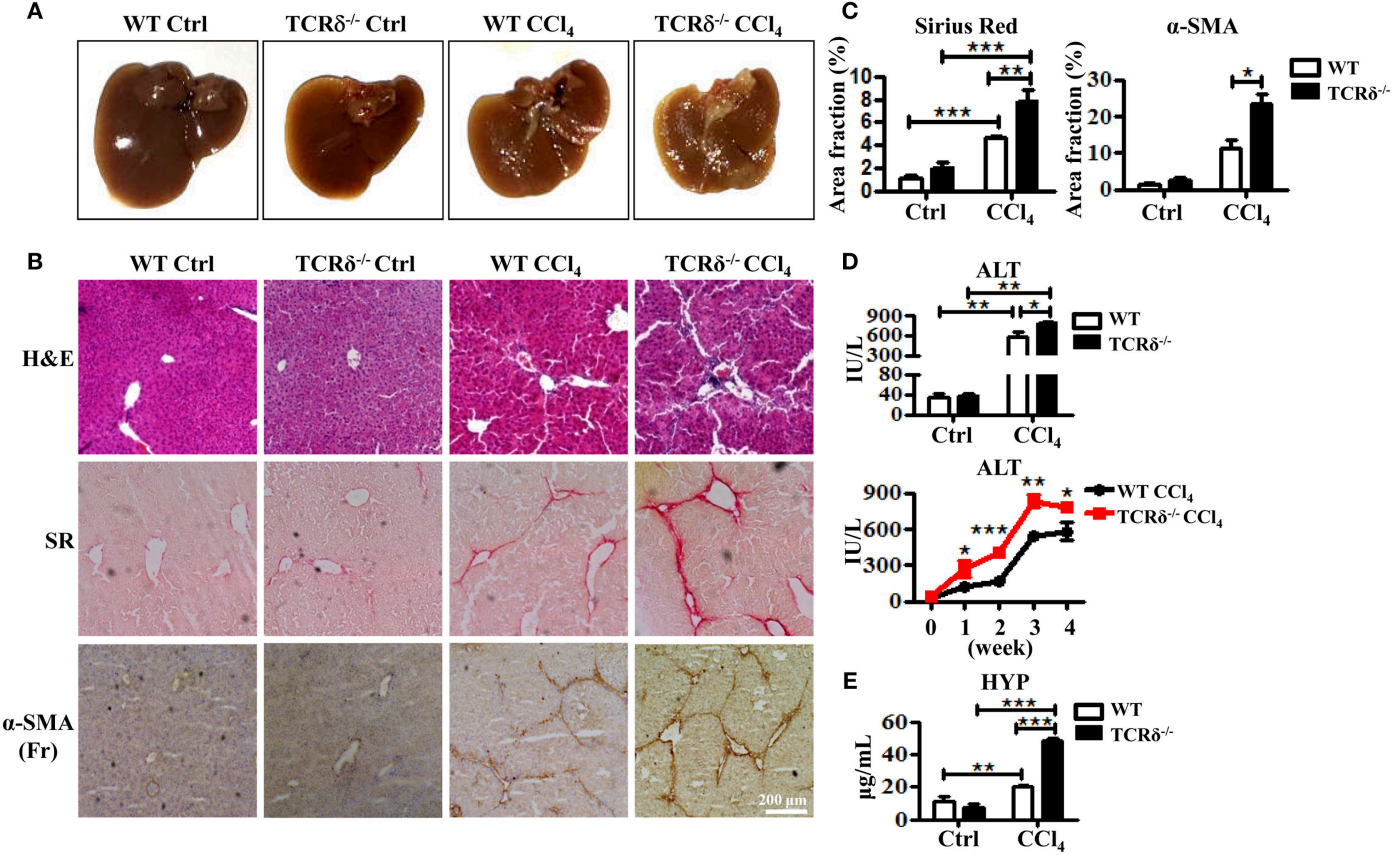
TCRδ^−/−^ mice develop more severe hepatic fibrosis upon chronic liver injury. Liver fibrosis was induced in WT and TCRδ^−/−^ mice by intraperitoneal injection of CCl_4_ (0.6 ml/kg) three times per week for 4 weeks. **(A)** Representative photograph of livers from WT and TCRδ^−/−^ mice treated with CCl_4_ or corn oil [control (Ctrl)]. **(B)** Representative H&E and Sirius Red (SR) staining of frozen liver sections from WT and TCRδ^−/−^ mice, and immunohistochemistry (IHC) for α-SMA in the livers of WT and TCRδ^−/−^ mice. Scale bar: 200 μm. **(C)** Quantification of matrix deposition from SR staining (left) and expression of α-SMA (right) as shown in **(B)**. Data are shown as mean ± SEM (*n* = 4). **(D)** Serum ALT levels in WT and TCRδ^−/−^ mice at 48 h after the last CCl_4_ injection (top) and at the indicated time points (bottom). **(E)** Serum hydroxyproline (HYP) contents were measured by ELISA. ^*^P < 0.05, ^**^P < 0.01, ^***^P < 0.001.

To investigate the role of γδT cells in chronic liver diseases, we established a murine model of liver fibrosis in WT and TCRδ^−/−^ mice via intraperitoneal injection of CCl_4_. The TCRδ^−/−^ mice displayed more severe liver fibrosis than the WT mice (Figures 1A,B). Hematoxyin and eosin (H&E) stained liver tissue sections showed more severely damaged liver architecture and more significant inflammatory infiltration in the livers of TCRδ^−/−^ mice than in those of the WT mice (Figure 1B). Sirius Red (SR) staining showed increased extracellular collagen deposition, immunohistochemical (IHC) staining demonstrated higher expression of α-SMA, a marker of activated HSCs further confirming the markedly increased liver fibrosis in TCRδ^−/−^ mice compared with WT mice (Figures 1B,C). Immunofluorescence staining for α-SMA and desmin also revealed more significant activation of HSCs in TCRδ^−/−^ mice than in WT mice after repeated CCl_4_ injections (Supporting Figures 2A,B). In addition, the fibrosis process was shown accompanied by elevated levels of serum ALT and hydroxyproline (HYP) (Figures 1D,E). And there were more significant intrahepatic CD45^+^leukocyte accumulation in CCl_4_-treated TCRδ^−/−^ mice compared with CCl_4_-treated WT mice Supporting Figure 2C), suggesting that missing γδT cells might promote the infiltration of inflammatory cells into liver tissue and then lead to the death of hepatocytes. These data strongly suggested that γδT cells have a protective role during CCl_4_-induced liver fibrosis.

### γδT Cells Recruit and Co-localize With HSCs in the Fibrotic Liver and Promote HSCs Apoptosis

**Figure 2:**
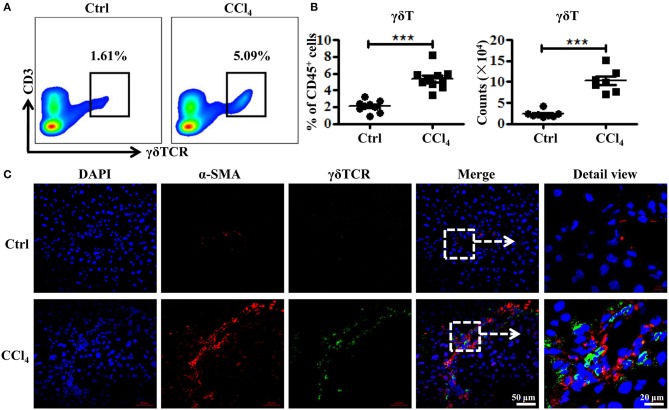
Increased γδT cells infiltration and their co-localization with activated HSCs in fibrotic livers. **(A)** Representative FACS plots of γδT cells in livers of WT mice treated with CCl_4_ or corn oil [control (Ctrl)]. **(B)** (Left) Statistical analysis of the percentage of γδT cells in CD45^+^ leukocytes. (Right) Absolute cell number of hepatic γδT cells. Data are shown as mean ± SEM. **(C)** Liver sections (frozen) of WT mice treated with CCl_4_ or corn oil (Ctrl) were stained for α-SMA to identify activated HSCs (red) and γδTCR to identify γδT cells (green). Nuclei were counterstained with DAPI (blue). An arrow indicates the detailed view of indicated area. ^***^P < 0.001.

To investigate how γδT cells prevent liver fibrosis during CCl_4_-induced chronic liver injury, we analyzed the changes in the frequency and numbers of liver γδT cells using flow cytometry following fibrosis. The proportion of γδT cells increased significantly from 2.09 ± 0.24 to 5.38 ± 0.40% after repeated CCl_4_ administration compared with the controls (Figures 2A,B). The absolute numbers of γδT cells in liver tissues also markedly increased (Figure 2B). Thus, chronic inflammation might promote the recruitment of γδT cells. HSCs are the major contributor to the formation of fibrosis because of their ability to transdifferentiate into collagen-producing myofibroblasts (22). Therefore, we hypothesized that the accumulated γδT cells might interact with activated HSCs during CCl_4_-induced liver fibrosis. We analyzed the distribution of γδT cells and activated HSCs in fibrotic liver using bi-color immunofluorescence staining for γδT cells (γδTCR) and activated HSCs (α-SMA). We observed larger numbers of γδTCR^+^ T cells (green) accumulated in the fibrotic livers, and these γδT cells were located close to activated HSCs (α-SMA^+^, Red) in the periportal region and fibrotic septa during liver fibrosis (Figure 2C), which suggested that these infiltrating γδT cells might directly interact with activated HSCs.

**Figure 3:**
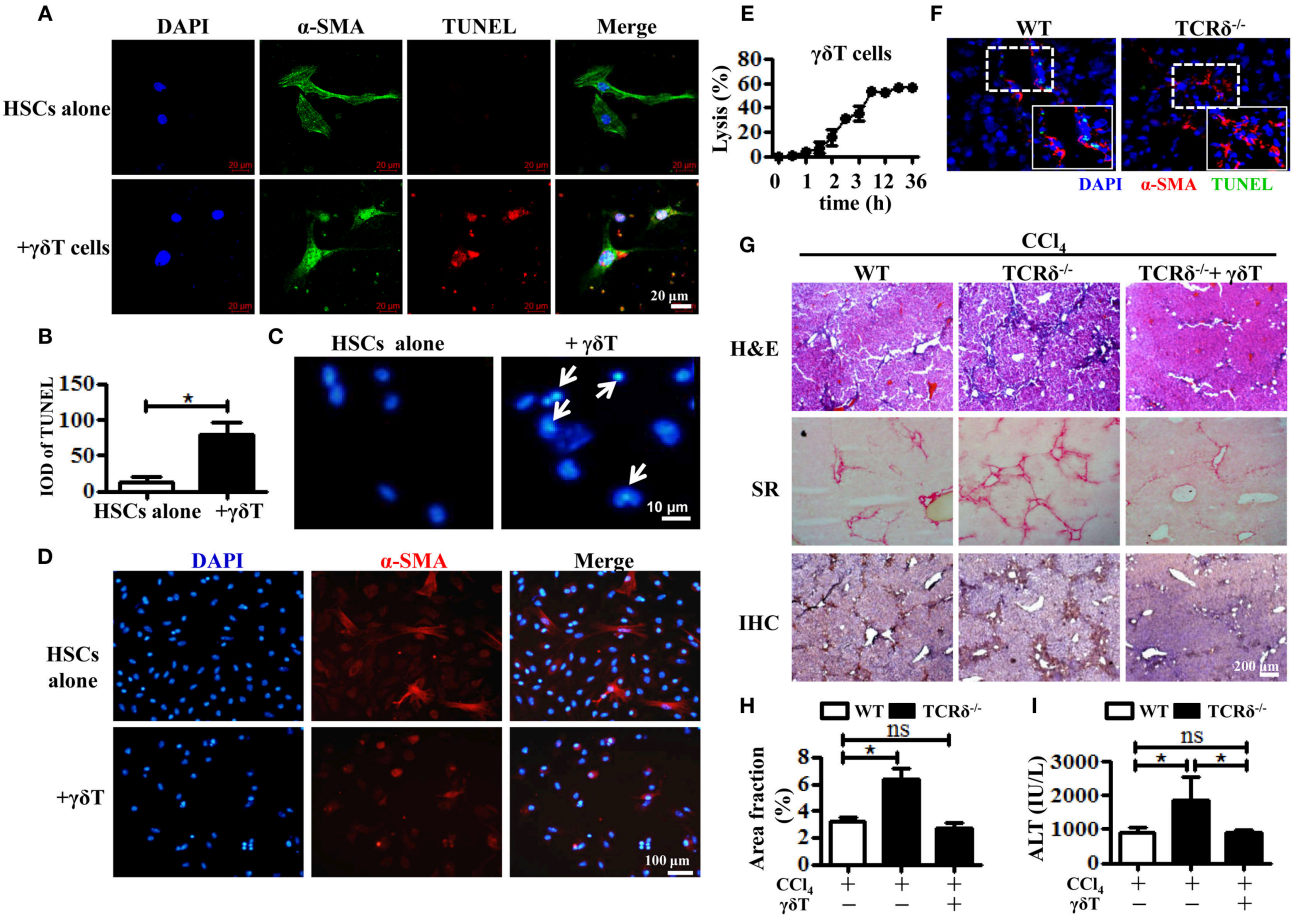
Hepatic γδT cells induce apoptosis of activated HSCs and attenuate liver fibrosis. **(A)** The primary HSCs were isolated from WT mice and seeded on 24-well plates for 5 days, and then co-cultured with isolated hepatic γδT cells from CCl_4_-treated WT mice. HSCs were stained with anti-α-SMA antibodies (green) and TUNEL (red) to identify apoptotic cells. Nuclei were counterstained with DAPI (blue). **(B)** Quantification of the expression of TUNEL as shown in **(A)**. **(C)** DAPI staining for apoptotic nuclei of activated HSCs after co-culturing with or without γδT cells. The HSCs co-cultured with γδT cells (+ γδT cells) showed apoptosis characterized by the chromatin becoming highly coacervate, nuclear envelope breakdown, and the production apoptotic bodies. An arrow indicates a representative apoptotic HSCs. **(D)** Immunofluorescence staining for α-SMA in activated HSCs after co-culturing with hepatic γδ T cells for 24 h (+ γδ T) or activated HSCs alone (HSCs alone). **(E)** Activated **HSCs** were incubated with or without γδT cells at an effector:target ratio of 2:1 for 36 h. The time-lapse lysis (%) was calculated by counting the numbers of adherent live HSCs at different time points. **(F)** Frozen sections of livers from fibrotic WT and TCRδ^−/−^ mice were stained with TUNEL (green) and anti-α-SMA antibody (red), scale bar: 50 μm. The dotted area in the slide was enlarged and shown in the inset. **(G)** Representative H&E staining, SR staining, and IHC for α-SMA of liver sections. Scale bar: 200 μm. **(H)** Quantification of matrix deposition from SR staining as shown in **(G)**. **(I)** Serum ALT levels in CCl_4_-treated WT mice and TCRδ^−/−^ mice after adoptively transferring hepatic γδT cells or normal saline in the same volume. ^*^P < 0.05.

Consequently, we hypothesized that the accumulated γδT cells could prevent liver fibrosis by killing activated HSCs. We first observed the role of hepatic γδT cells in inducing the apoptosis of activated HSCs by co-culturing isolated hepatic γδT cells with culture-activated HSCs, and then detecting HSCs apoptosis in frozen liver section from CCl_4_-treated WT and TCRδ^−/−^ mice. The primary HSCs were isolated from WT mice by density gradient centrifugation and cultured in 24-well culture plates to induce culture-activated HSCs (23). Freshly isolated primary HSCs were non-proliferating (quiescent), retinoid-storing cells with autofluorescence after ultraviolet excitation, which allowed their identification. Thus, the purity of the isolated HSCs was determined by detecting the autofluorescence under ultraviolet light. Quiescent HSCs on the plates will lose vitamin A droplets and transdifferentiate into a myofibroblast-like phenotype that mimic the process of activation that is thought to occur *in vivo* (24). As shown in Supporting Figure 3A, almost all of freshly isolated primary HSCs exhibited autofluorescence under ultraviolet light. And increased expression of α-SMA was observed from the 3rd day and sustained for at least 18 days after isolation Supporting Figures 3B,C). Then we co-cultured these culture-activated HSCs with freshly-isolated hepatic γδT cells from CCl_4_-treated WT mice. Immunofluorescence double staining for α-SMA and TUNEL was performed to assess HSC apoptosis. The activated HSCs were shown undergo apoptosis after co-culturing with γδT cells (Figures 3A,B). The nuclei of HSCs co-cultured with γδT cells also displayed representative apoptotic features, such as chromatin condensation and margination (Figure 3C). We also observed that hepatic γδ T cells may induce the inactivation of activated HSCs, as evidenced by the decreased expression of α-SMA in HSCs after co-culturing with hepatic γδ T cells (Figure 3D). In addition, significant cytolytic activity of γδT cells against activated HSCs were detected at different time points (Figure 3E). These results suggested that hepatic γδT cells could directly kill activated HSCs.

To confirm this effect *in vivo*, we performed immunofluorescent double staining for α-SMA and TUNEL in frozen liver sections from fibrotic models of WT and TCRδ^−/−^ mice. Loss of γδT cells significantly inhibited the apoptosis of activated HSCs in fibrotic livers, consistent with the *in vitro* results (Figure 3F). Then, hepatic γδT cells isolated from WT mice were transferred into TCRδ^−/−^ mice once per week during the process of treating with repetitive CCl_4_ injections (Supporting Figures 4A–C), according to a previous reported method (8). Severe fibrosis and liver damage were markedly alleviated after transferring hepatic γδT cells from WT mice, as displayed by reduced extracellular collagen deposition and serum ALT levels (Figures 3G–I). These data confirmed the protective effects of γδT cells by directly killing activated HSCs or inducing inactivation of activated HSCs in CCl_4_-induced liver fibrosis.

### Chronic Inflammation Promotes Hepatic γδT Cells Expression of NKp46, Which Is Involved in the Enhanced Killing of Activated HSCs by γδT Cells

**Figure 4:**
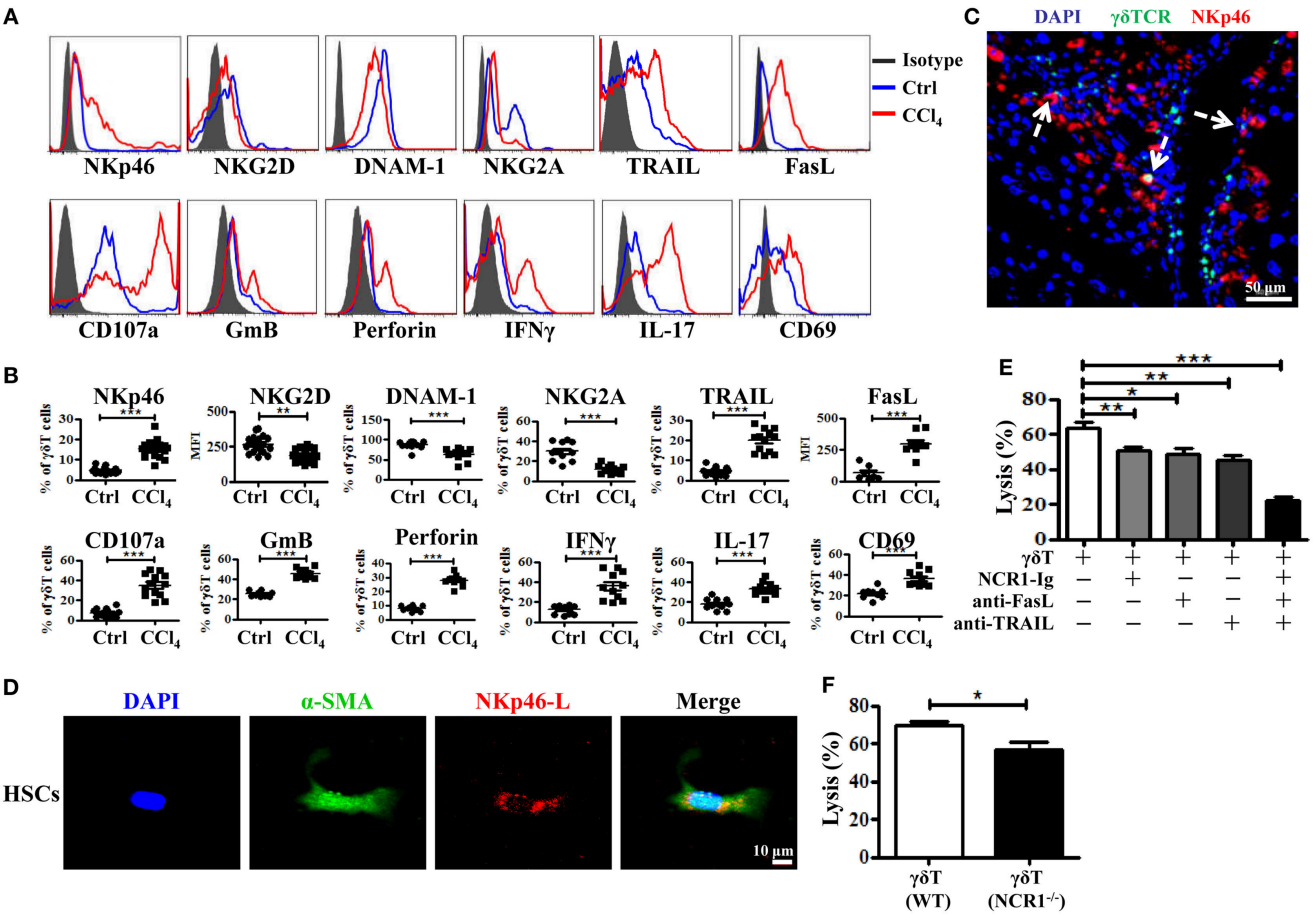
Hepatic γδT cells exhibit strong cytotoxic potential upon chronic liver injury. **(A)** Representative histograms of NKp46, NKG2D, DNAM-1, NKG2A, TRAIL, FasL, CD107a, GmB, perforin, IFNγ, IL-17, CD69 expression on hepatic γδT cells from WT mice treated with corn oil (Ctrl) and CCl_4_. **(B)** The statistical percentages or mean fluorescence intensity (MFI) are shown (*n* = 10–15). **(C)** Representative immunofluorescence double staining image of frozen liver fibrotic sections (*n* = 3) stained for γδT cells (γδTCR^+^, green) and NKp46 (NKp46^+^, red) and counterstained with DAPI (blue). Scale bars, 50 μm. Arrows indicate γδTCR and NKp46 double positive cells. **(D)** Representative images of culture-activated HSCs (α-SMA^+^, green) *in vitro* which stained for NKp46-L with NCR1 Fc chimera (NCR1-Ig) (red) and counterstained with DAPI. Scale bars, 50 μm. **(E)** The primary HSCs were isolated from WT mice, 5 days after adherent culture, co-cultured with isolated hepatic γδT cells at an effector: target ratio of 2:1 in the presence or absence of NCR1-Ig, TRAIL-, or FasL-specific neutralizing antibodies for 12 h, respectively. The cytotoxicity of hepatic γδT cells against activated HSCs were measured using a lactate dehydrogenase (LDH) assay. **(F)** The hepatic γδT cells isolated from CCl_4_-treated WT and NCR1^−/−^ mice were co-cultured with activated HSCs at an effector:target ratio of 2:1 for 12 h, the cytotoxicity was measured using an LDH assay. ^*^P < 0.05, ^**^P < 0.01, ^***^P < 0.001.

γδT cells express certain natural killer receptors, such as NKG2D, DNAX accessory molecule-1 (DNAM-1), and natural cytotoxicity receptors (NCR) NKp30 and NKp44, which are involved in the activation and cytolytic capacity of γδT cells (25). We investigated whether chronic inflammation during liver fibrosis changed the expression levels of these receptors on γδT cells. CCl_4_-induced chronic inflammation significantly increased the expression of NKp46, but decreased the expression levels of NKG2D, DNAM-1, and NKG2A on hepatic γδT cells (Figures 4A–C and Supporting Figures 5A–C). Although NKG2D and DNAM-1 exhibited lower expression upon chronic liver injury, hepatic γδT cells exhibited strong cytotoxic potential upon chronic liver injury, as evidenced by the higher expression of CD107a, GmB, and perforin (Figures 4A,B). Therefore, we proposed that the higher expression of NKp46 and the lower expression of NKG2A contributed to the strong cytotoxic potential of γδT cells in CCl_4_-induced liver fibrosis. Noticeably, NKp46 is the only NCR found in mice (26). Interestingly, we observed that hepatic γδT cells could acquire NKp46 expression following CCl_4_-induced liver fibrosis (Figures 4A,B). Immunofluorescence double staining for γδTCR and NKp46 further confirmed that hepatic γδT cells express NKp46 in fibrotic livers (Figure 4C). Most hepatic γδT cells in fibrotic livers expressed NKp46, but those in normal livers barely expressed NKp46 (Supporting Figures 5A–C).

We then confirmed whether γδT cells kill activated HSCs through NKp46-mediated cytotoxicity. First, we tested whether the activated HSCs expressed the ligand of NKp46 (NKp46-L). The ligands for mouse NCR1 are unknown; therefore, we stained the activated HSCs with an NCR1-Ig fusion protein. As shown in Figure 4D, activated HSCs (α-SMA^+^) could be stained by NCR1-Ig, which indicated that activated HSCs expressed NKp46L. We then assessed the lytic functions of γδT cells against activated HSCs in the presence of NCR1-Ig, which could block the binding between NKp46 on γδT cells and NKp46L on the activated HSCs. We found that NCR1 blockade significantly attenuated γδT cell- mediated lysis of activated HSCs (Figure 4E). In addition, hepatic γδT cells derived from NCR1^−/−^ mice also exhibit impaired lytic functions compared with those from WT mice (Figure 4F). Thus, we concluded that NKp46 is the predominant receptor involved in killing HSCs during chronic liver injury.

Notably, the lysis ability of γδT cells against activated HSCs was only partly decreased following NKp46 blockade, suggesting that other factors are involved in the cytotoxicity of γδT cells against HSCs. Death receptor-mediated apoptosis is involved in the cytotoxicity of NK cells or T cells (27). Figures 4A,B showed that hepatic γδT cells from CCl_4_-induced fibrotic mice expressed higher levels of TRAIL and FasL than those isolated from control mice, which suggested that hepatic γδT cells might induce the apoptosis of activated HSCs in a TRAIL- or FasL-dependent manner. To test this, we compared the lytic functions of γδT cells against activated HSCs in the presence of FasL-specific and TRAIL-specific blocking antibodies, respectively. Figure 4E showed that blockade of TRAIL and FasL significantly attenuated the lytic ability of γδT cells against activated HSCs. However, NKp46 blockade, FasL blockade, or TRAIL blockade, separately, only partly inhibited the lysis ability of γδT cells against activated HSC, while blocking all three simultaneously caused extreme attenuation of γδT cells-mediated lysis (Figure 4E). Taken together, our results revealed that hepatic γδT cells exert their lytic functions against activated HSCs in NKp46-, TRAIL-, and FasL-dependent manner.

### IFNγ-Producing γδT Cells Exhibit More Significant Protection Upon Chronic Liver Injury

**Figure 5:**
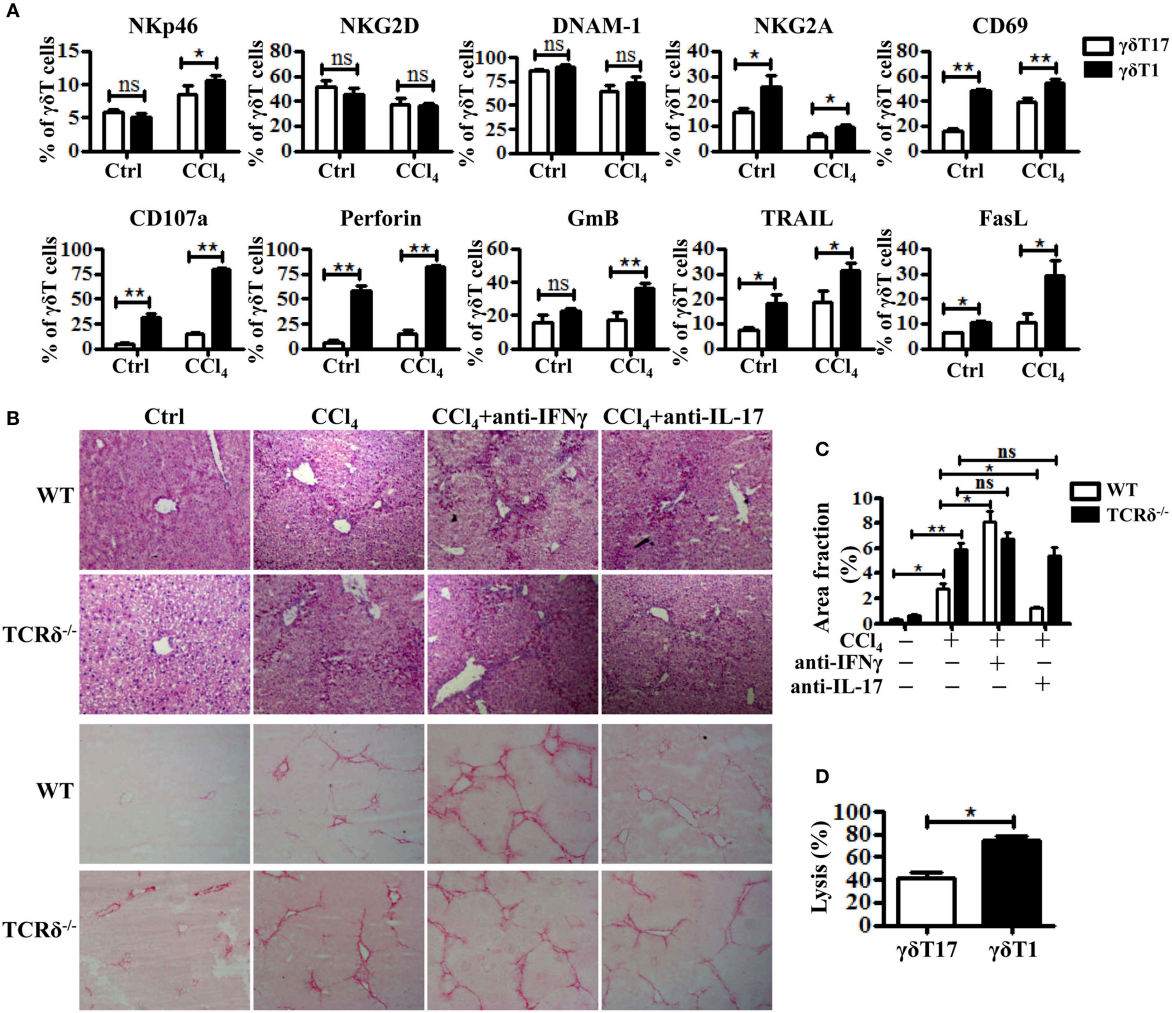
Hepatic γδT subpopulations exhibit distinct anti-fibrotic ability upon chronic liver injury. **(A)** The levels of NKp46, NKG2D, DNAM-1, NKG2A, CD69, CD107a, Perforin, GmB, TRAIL, and FasL on hepatic γδT17 cells and γδT1 cells were determined by FACS. **(B)** Representative H&E and SR staining of frozen liver sections from WT mice treated with corn oil [control (Ctrl)], CCl_4_, CCl_4_ following IFNγ-blockade (CCl_4_+anti-IFNγ), or CCl_4_ following IL-17A blockade (CCl_4_+anti-IL-17A), respectively. **(C)** Quantification of matrix deposition from SR staining as shown in **(B)**. **(D)** The hepatic γδT17 cells and γδT1cells from CCl_4_-treated WT mice were co-cultured with activated HSCs at an effector:target ratio of 2:1, respectively. The cytotoxicity of both hepatic γδT17 cells and γδT1 cells against activated HSCs were measured using a LDH assay. ^*^P < 0.05, ^**^P < 0.01.

Based on their cytokine production, γδT cells are usually divided into two subsets: IFNγ-producing γδT (γδT1) cells and IL17-producing γδT (γδT17) cells (7, 28). The two subsets might have different roles in the pathogenesis of liver diseases, but have not been clarified. As shown in Figures 4A,B, hepatic γδT cells produced high levels of IFNγ and IL-17 upon chronic liver injury. Through comparing the expression levels of receptors and effector molecules on the two γδT subsets, we found that γδT1 cells exhibited higher expression levels of NKp46, CD107a, GmB, perforin, TRAIL, and FasL than γδT17 cells upon chronic liver injury (Figure 5A). We next examined whether IFNγ or IL-17 is responsible for the development of liver fibrosis by comparing the degree of liver fibrosis upon *in vivo* blockade of IFNγ and IL-17A in WT and TCRδ^−/−^ mice mice. H&E staining and SR staining showed more severe damage to the liver architecture and increased extracellular collagen deposition in IFNγ-blockade WT mice, while there was minor remission in IL-17A-blockade WT mice compared with non-blockade WT mice following fibrosis, and neither IFNγ nor IL-17A blockade affected fibrosis in TCRδ^−/−^ mice (Figures 5B,C). Further, *in vitro* killing assay showed that γδT1 cells exhibited higher cytotoxicity against activated HSCs than γδT17 cells (Figure 5D). These results suggested that IFNγ exerts a protective role during the development of liver fibrosis and that hepatic γδT cells, especially γδT1 subset, play a significant protective role during the development of liver fibrosis.

### γδT Cells Promote the Anti-fibrotic Ability of cNK and Lrnk Cells by Enhancing Their Cytotoxicity Against Activated HSCs

**Figure 6:**
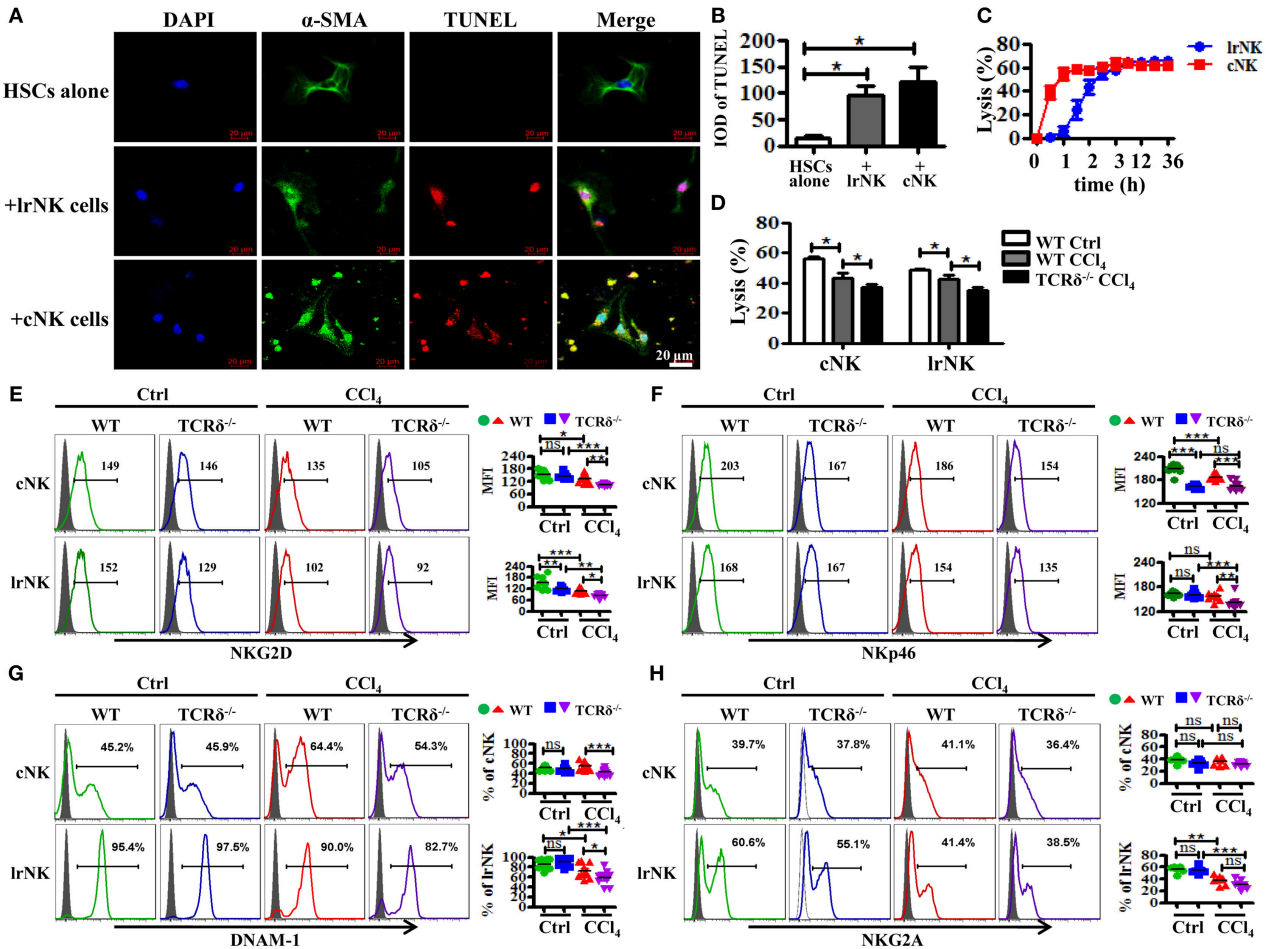
γδT cells deficiency attenuates the anti-fibrotic functions of both lrNK and cNK cells. **(A)** Culture-activated HSCs after co-culturing with lrNK cells (+lrNK) or cNK cells (+cNK) were stained with anti-α-SMA antibodies (green) and TUNEL (red) to identify apoptotic cells. Nuclei were counterstained with DAPI (blue). **(B)** Quantification of the expression of TUNEL as shown in **(A)**. **(C)** Activated HSCs were incubated with lrNK cells or cNK cells at an effector:target ratio of 2:1 for 36 h, respectively. The time-lapse lysis (%) were calculated by counting the numbers of adherent live HSCs at different time points. **(D)** Hepatic cNK cells and lrNK cells isolated from corn oil-treated WT mice (WT Ctrl), CCl_4_-treated WT mice (WT CCl_4_), and CCl_4_-treated TCRδ^−/−^ mice (TCRδ^−/−^ CCl_4_) were co-cultured with activated HSCs at an effector:target ratio of 2:1, respectively. The cytotoxicity of hepatic cNK cells and lrNK cells against activated HSCs was measured using a LDH assay. **(E–H)** (Left) Representative histograms of NKG2D, NKp46, DNAM-1, and NKG2A expression on cNK cells and lrNK cells were determined by **FACS**. (Right) Statistical analysis is shown, *n* = 9–10. ^*^P < 0.05, ^**^P < 0.01, ^***^P < 0.001.

γδT cells are able to interact with other immune cells upon activation (9, 29). NK cells, particularly cNK cells, inhibit the development of fibrosis by killing activated HSCs in a TRAIL-dependent manner (11). However, the role of lrNK cells, with high TRAIL expression, in fibrosis is unknown. Therefore, we investigated whether γδT cells influenced the progress of liver fibrosis indirectly by interacting with cNK and lrNK cells. Through *in vitro* co-culture experiments, we observed that both cNK and lrNK cells could induce the apoptosis of HSCs and efficiently kill activated HSCs (Figures 6A–C); therefore, we investigated whether γδT cells are responsible for regulating liver fibrogenesis by crosstalk with lrNK or cNK cells. We compared the percentages and absolute numbers of total NK, lrNK, and cNK cells in livers of WT and TCRδ^−/−^ mice following fibrosis, and found that they did not differ between WT mice and TCRδ^−/−^ mice (Supporting Figures 6A–D). However, the percentages and absolute numbers of lrNK cells were significantly lower in fibrotic livers from CCl_4_-treated TCRδ^−/−^ mice compared with those in CCl_4_-treated WT mice, while the percentages and absolute numbers of hepatic cNK cells were significantly increased in CCl_4_-treated TCRδ^−/−^ mice compared with those in CCl_4_-treated WT mice, which demonstrated that γδT cell deficiency altered the composition of NK cell subpopulations (Supporting Figures 6A–D).

We further compared the cytotoxicity of hepatic cNK cells and lrNK cells against activated HSCs from CCl_4_-treated WT mice compared with corn oil-treated WT mice. Both cNK and lrNK cells from fibrotic livers exhibited impaired cytolytic ability (Figure 6D). To further investigate whether γδT cells affect the anti-fibrotic effects of cNK and lrNK cells in fibrotic livers, we isolated hepatic cNK and lrNK cells from CCl_4_-treated WT mice or TCRδ^−/−^ mice, and then detected their cytotoxicity against activated HSCs. Both hepatic cNK and lrNK cells from CCl_4_-treated TCRδ^−/−^ mice exhibited markedly lower lysis ability against activated HSCs, compared to those from CCl_4_-treated WT mice (Figure 6D), suggesting that loss of γδT cells impaired the cytolytic ability of both cNK and lrNK cells.

NK cell responses are governed by a balance between activating and inhibitory receptors; therefore, we examined the receptor expression on lrNK and cNK cells from WT or TCRδ^−/−^ mice following fibrosis. Loss of γδT cells strongly limited the expression of NK cell killer activating receptors (NKG2D, NKp46, DNAM-1) on both lrNK and cNK cells upon chronic liver injury (Figures 6E–G); however, it barely affected the expression of NK cell inhibitory receptor, NKG2A (Figure 6H).

**Figure 7:**
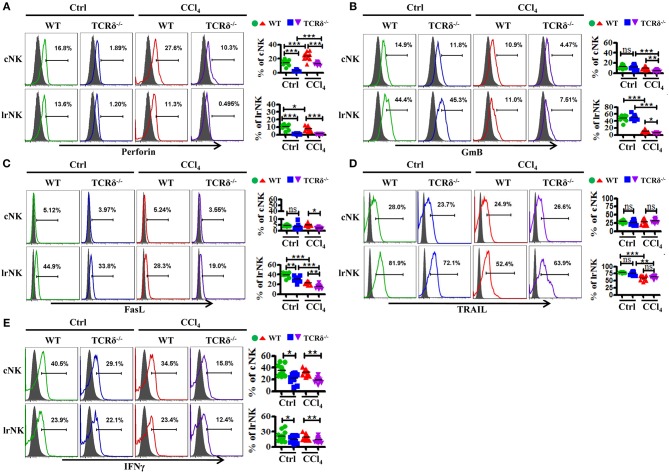
γδT cells deficiency impairs the expression of cytotoxic effector molecules of lrNK and cNK cells upon chronic liver injury. **(A–E)** Liver mononuclear cells were isolated from WT or TCRδ^−/−^ mice with or without CCl_4_ continuous administration, respectively. (Left) The expression of Perforin, GmB, FasL, TRAIL, and IFNγ on cNK cells and lrNK cells were determined by FACS. (Right) Statistical analysis is shown, *n* = 9–12. ^*^P < 0.05, ^**^P < 0.01, ^***^P < 0.001.

We then examined levels of cytotoxic effector molecules in cNK and lrNK cells. The levels of perforin, GmB and FasL as well as the production of IFNγ in hepatic cNK and lrNK cells decreased significantly in CCl_4_-treated TCRδ^−/−^ mice compared with those in CCl_4_-treated WT mice (Figures 7A–C,E). However, the expression of TRAIL unchanged (Figure 7D). Taken together, these results indicated that γδT cells might promote the functions of lrNK and cNK cells by enhancing NK cell activation and augmenting the expression of cytotoxic effector molecules (perforin, GmB, FasL, and IFNγ).

### Crosstalk Between γδT Cells and cNK Cells as Well as lrNK Cells

**Figure 8:**
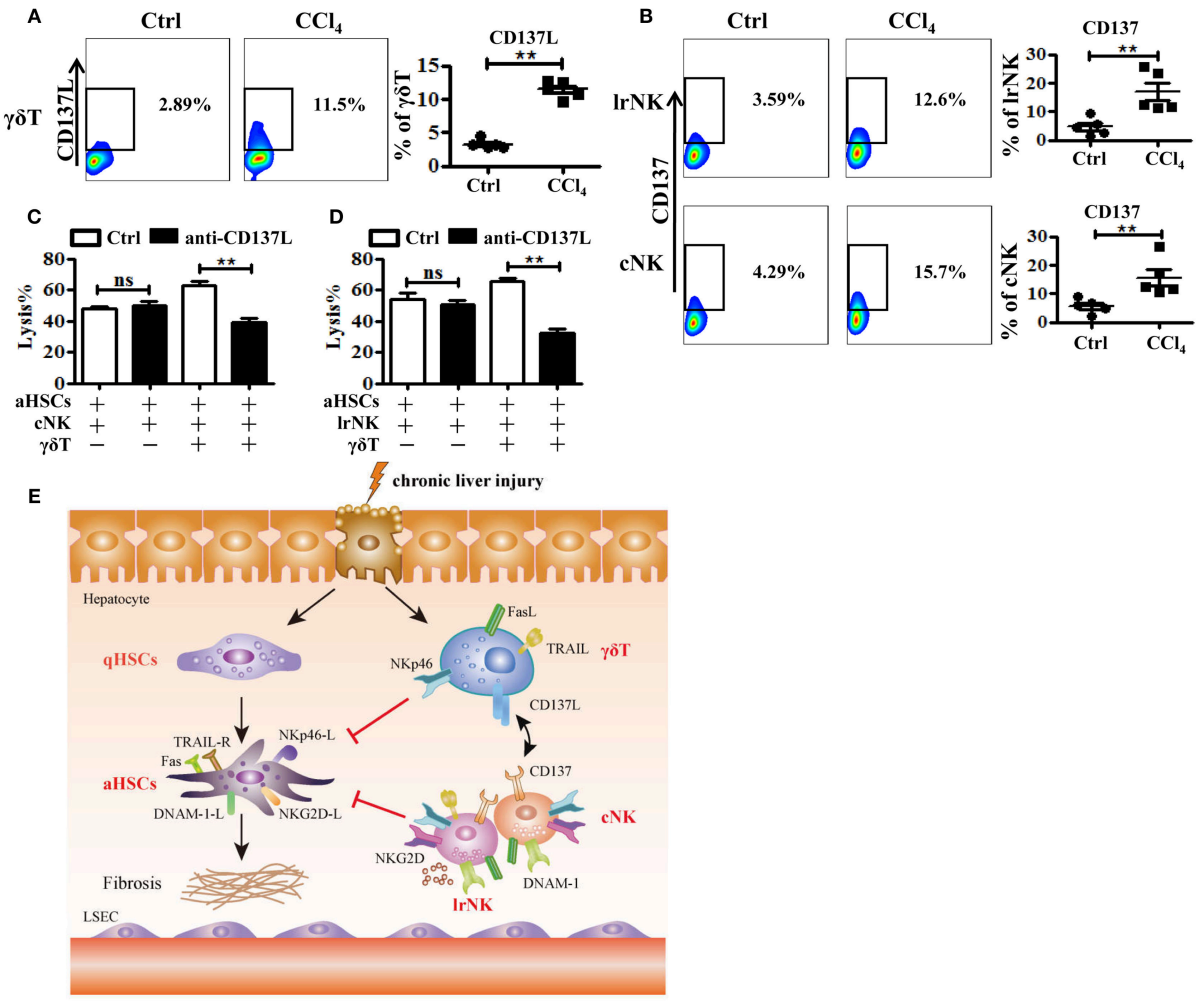
The crosstalk between γδT cells and cNK cells as well as lrNK cells. **(A)** Liver mononuclear cells were isolated from WT mice with or without CCl_4_ continuous administration, respectively. (Left) The expression of CD137 ligand (CD137L) on hepatic γδT cells was determined by FACS. (Right) Statistical analysis is shown, *n* = 6. **(B)** (Left) The expression CD137 on hepatic cNK and lrNK cells was determined by FACS, respectively. (Right) Statistical analysis is shown, *n* = 6. **(C, D)** The culture-activated HSCs were co-cultured with isolated hepatic lrNK cells or cNK cells with/without γδT cells at an effector:target ratio of 2:1 in the present or absence of CD137L-specific blocking antibody (anti-CD137L) for 12 h, respectively. The cytotoxicity against activated HSCs was determined using LDH assay. **(E)** Schematic model for the anti-fibrotic functions of hepatic γδT cells in CCl_4_-induced liver fibrosis, through directly killing against activated HSCs dependent on NKp46-mediated cytotoxicity, and increasing NK cells-mediated cytotoxicity against activated HSCs through CD137-CD137L interaction in CCl_4_-induced liver fibrosis. ^**^P < 0.05.

There is a crosstalk between γδT cells and NK cells, by which γδT cells increase NK cell-mediated cytotoxicity against tumor via engagement of the co-stimulatory receptor 4-1BB (CD137) (9). To further investigate how γδT cells promote the anti-fibrotic effects of lrNK and cNK cells in CCl_4_-induced liver fibrosis, we tested whether CD137 participates in the regulation of NK cell effector activities in fibrotic livers. We assessed the expression of CD137's ligand (CD137L) on hepatic γδT cells and the expression of CD137 on cNK and lrNK cells upon chronic liver injury. The expression of CD137L on hepatic γδT cells increased after liver fibrosis (Figure 8A). Correspondingly, the levels of CD137 on lrNK and cNK cells also increased in the liver fibrosis model (Figure 8B). Then, we compared the cytotoxicity of hepatic cNK or lrNK cells co-cultured with γδT cells from fibrotic livers against activated HSCs in the presence or absence of a CD137L-specific blocking antibody. We found that CD137 blockade did not impair the direct lytic functions of hepatic cNK and lrNK cells against HSCs; however, in the cNK or lrNK and γδT cell co-culture system, the increased lytic functions of hepatic cNK and lrNK cells against activated HSCs promoted by γδT cells were significantly attenuated in the presence of the CD137L-specific blocking antibody (Figures 8C,D), which suggested that the CD137-CD137L interaction plays a major role in the process of γδT cells' contribution to the cytotoxicity of cNK and lrNK cells against activated HSCs.

## Discussion

The roles of γδT cells in fibrogenesis remain incompletely understood. Some studies revealed that γδT cells might inhibit fibrogenesis by inducing apoptosis of activated HSCs in a FasL-dependent manner, while others emphasized the pathogenic role of γδT cells through IL-17A-mediated enhancement of HSCs activation in CCl_4_-induced liver injury and liver fibrosis (8, 19, 30). In the present study, we demonstrated the key role of γδT cells in the modulation of liver fibrosis, using a CCl_4_-induced liver fibrosis model in WT and TCRδ^−/−^ mice. Our data revealed that γδT cells attenuate fibrogenesis by directly killing activated HSCs via NKp46-mediated release of cytolytic granules and death receptor-mediated apoptosis. Moreover, we confirmed that γδT cells also enhance the anti-fibrotic effects of lrNK and cNK cells, partly dependent on the CD137-CD137L interaction (Figure 8E).

Natural killer receptors were initially described as NK cell-specific receptors, including lectin-type receptors, NCRs, and killer immunoglobulin receptors, and the balance between activating and inhibitory signals controls NK cell functionality (31, 32). However, some natural killer receptors (e.g., NKG2D and DNAM-1) are expressed by γδT cells. Particularly, NKG2D is constitutively expressed on most γδT cells and plays critical roles in discriminating stressed cells (transformed or infected) from healthy cells (33, 34). Notably, γδT cells usually do not express NCRs under normal conditions, but acquire the expression of NCRs, such as NKp30 and NKp44, to enhance their effector function during viral infection or tumorigenesis (33). For instance, NKp30 and NKp44 are highly induced on Vδ1^+^T cells after continued activation by γδTCR stimulation in the presence of IL-2 or IL-15, and the inducible NCRs endows Vδ1^+^T cells with enhanced cytotoxicity against leukemia cells and increased ability to control of virus infection (35, 36). Although NKp46 can also be induced on Vδ1^+^T cells, they do not enhance Vδ1^+^T cells-mediated cytotoxicity against tumor cells (35). As the only NCR found in mice, the enigmatic role of inducible NKp46 on γδT cells remains unclear. To date, there has been no report on the expression of NCR on γδT cells during chronic inflammation-induced liver injury or liver fibrosis. In the present study, we found that CCl_4_ stimulation-induced chronic liver injury induces the expression of NKp46 on activated γδT cells, which was confirmed by flow cytometry and immunofluorescence staining (Figures 4A–C and Supporting Figure 5). In addition, chronic inflammation stimulated the activated HSCs to express NKp46L (Figure 4D). The acquisition of NKp46 significantly promoted the cytolytic activity of γδT cells against HSCs, as confirmed by NCR1 blocking and in NCR1^−/−^ mice (Figures 4E,F). Although TRAIL and FasL also contributed to the enhanced cytotoxicity of γδT cells against HSCs, this is the first report of the inducible expression of NKp46 on hepatic γδT cells and its critical role in enhancing the activation and function of γδT cells during chronic inflammation.

γδT cells show phenotypic and functional heterogeneity and comprises diverse subsets with distinct anatomical locations and distinct functional properties (37, 38). The contradictory roles of γδT cells in liver fibrosis might be associated with the roles of these different subsets (39). Based on their cytokine production, γδT cells are usually divided into two subsets: γδT1 and γδT17 cells (40). Human γδT cells are usually divided into two major structural subsets according to their TCRδ chain usage: Vδ1 and Vδ2 T cells (28, 41). The majority of liver resident γδT cells in human chronic liver disease were Vδ2^−^ γδT cells, which mainly produce cytokines IFNγ and TNFα (42). Murine γδT cells are usually divided into 7 structural subsets (Vγ1–Vγ7) based on their Vγ usage (43). We have detected the murine hepatic γδT subset distribition and found that murine liver mainly composed of two main subsets: Vγ1^+^γδT and Vγ4^+^γδT subsets (data not shown). It is reported that hepatic Vγ1^+^γδT cells mainly produce IFNγ and TNFα, which is consistent with Vδ2^−^ γδT cells in human chronic liver disease, while hepatic Vγ4^+^γδT cells produce both IL-17 and IFNγ (43, 44). In the present study, we compared the different effects of γδT1 and γδT17 in CCl_4_-induced liver fibrosis and found that γδT1 cells express higher levels of NKp46 and cytolytic effector molecules, and thus exert stronger cytotoxic potential against activated HSCs than γδT17 cells. IFNγ seems to exert a markedly anti-fibrotic ability, characterized by more severe fibrosis in CCl_4_-treated IFNγ-blockade mice, compared with that in CCl_4_-treated WT mice (Figure 5). Although IL-17A was implicated in promoting liver fibrosis by inducing HSCs activation (30), we only observed a slight decrease in the degree of fibrosis during IL-17A blockade in CCl_4_-induced fibrosis. These results suggest that the anti-fibrotic effect of IFNγ overwhelms the IL-17A-mediated pro-fibrotic effect in CCl_4_-induced fibrosis. Considering the similarity of murine γδT1 or Vγ1^+^γδT cells and human Vδ2^−^ γδT cells, we proposed that our study may provide inspiration for the investigation of γδT cell-based therapies in patient cohorts.

γδT cells are believed to mediate or regulate immune-mediated diseases through interactions with other immune cells (45). NK cells were reportedly involved in inhibiting the development of fibrosis by killing activated HSCs. Particularly, cNK cells protect against liver fibrosis by killing activated HSCs in a TRAIL-dependent manner; however, the role of lrNK cells in fibrosis is unclear (11). In the present study, we demonstrated that cNK and lrNK cells exert anti-fibrotic effects by killing activated HSCs. However, CCl_4_-induced chronic inflammation impaired the antifibrotic capacity of NK cells (Figure 6D), which was consistent with a previous report (46). Notably, γδT cells enhanced the anti-fibrotic effects of lrNK and cNK cells, as evidenced by the decreased cytotoxity of both NK cell types against activated HSCs by from CCl_4_-treated TCRδ^−/−^ mice. γδT cells augment NK cell-mediated antitumor cytotoxicity through CD137 engagement (9). Our results demonstrated that γδT cells could increase the anti-fibrotic functions of cNK and lrNK cells through the CD137-CD137L interaction following fibrosis. To the best of our knowledge, this study is the first to describe crosstalk between γδT cells and NK cells during fibrosis development. However, CD137L blockade only partly, but not completely, inhibited the lytic ability of NK cells against activated HSCs in the presence of γδT cells suggesting that other factors may also contribute to the interaction between γδT cells and NK cells.

The preventive role of γδT cells in attenuating CCl_4_-induced liver injury is not only attributed to the direct and indirect killing of activated HSCs, but also possibly involved in the protection of hepatocytes. We observed that lack of γδT cells not only exacerbates liver fibrosis, but also resulted in elevated serum ALT levels, accompanied with more significant intraheptic leukocyte accumulation, compared to CCl_4_-treated WT mice (Supporting Figure 2C), suggesting that missing γδT cells might promote the infiltration of inflammatory cells to liver tissue and then lead to the death of hepatocytes. The results indicated that hepatic γδT cells might inhibit the infiltration of inflammatory cells to liver tissue during fibrosis, although the precise effect and mechanism need further investigation. In accordance with this opinion, Linda Hammerich's paper also found higher intrahepatic leukocytes infiltration when inhibiting accumulation of γδT cells, while the adoptive transfer of γδT cells reduced hepatic inflammation and fibrosis upon chronic injury in CCR6^−/−^ mice (8). In addition, the direct role of γδT cells on HSCs during liver fibrosis includes inducing both the apoptosis of HSCs and the inactivation of activated HSCs.

In summary, based on TCRδ^−/−^ mice, we identified the protective role of γδT cells by directly killing activated HSCs via NKp46-mediated cytotoxicity and TRAIL-and FasL-mediated apoptosis in a CCl_4_-induced liver fibrosis model. γδT1 cells play a major protective role during the process. In addition, γδT cells significantly enhanced NK cell-mediated cytotoxicty activity against activated HSCs in a co-stimulatory receptor CD137-dependent manner. Therefore, our study provides a detailed insight into the mechanisms of γδT cell-mediated anti-fibrotic effects and suggests valuable therapeutic targets to treat liver fibrosis.

## Author Contributions

ML designed and performed experiments, analyzed data, and wrote the manuscript. YH and YY performed experiments and analyzed data. ZT provided essential deficient mice, provided guidance, and suggestions for the study. CZ conceived and supervised the study, designed experiments, analyzed and interpreted data, and wrote the manuscript.

### Conflict of Interest Statement

The authors declare that the research was conducted in the absence of any commercial or financial relationships that could be construed as a potential conflict of interest.

## Abbreviations

HSCs: hepatic stellate cells
CCl_4_: carbon tetrachloride
WT: wild-type
TCRδ^−/−^: γδT cell deficient
γδT1: IFNγ-producing γδT cells
γδT17: IL-17-producing γδT cells
CD137: cotimulatory receptor 4-1BB
NK: natural killer
cNK: conventional NK cells
lrNK: liver-resident NK cells
TRAIL: tumor necrosis factor-related apoptosis-inducing ligand
CD137L: ligand of CD137
SR: Sirius red
α-SMA: α-smooth muscle actin
FasL: CD95 ligand
DNAM-1: DNAX accessory molecule-1
NCR: natural cytotoxicity receptors
GmB: granzyme B
TUNEL: Terminal deoxynucleotidyl transferase-mediated dUTP nick-end-labeling
DAPI: 2-(4-amidinophenyl)-1H-indole-6-carboxamidine
LMNCs: liver mononuclear cells
FACS: fluorescence activated cell sorting
ALT: aminotransferase activities
CFSE: carboxyfluorescein succinimidyl ester.
