# Supplementary material for: γδT Cells Suppress Liver Fibrosis via Strong Cytolysis and Enhanced NK Cell-Mediated Cytotoxicity Against Hepatic Stellate Cells
Source: Front Immunol. 2019 Mar 15;10:477. doi: 10.3389/fimmu.2019.00477 (PMC6428727; doi:10.3389/fimmu.2019.00477)
Supplement: Supplementary file 1 [file Data_Sheet_1.doc]

**γδT cells suppress murine liver fibrosis via strong cytolysis and enhanced NK cell-mediated cytotoxicity against hepatic stellate cells**

Meifang Liu, Yuan Hu, Yi Yuan, Zhigang Tian and Cai Zhang

**Supporting figures**

**
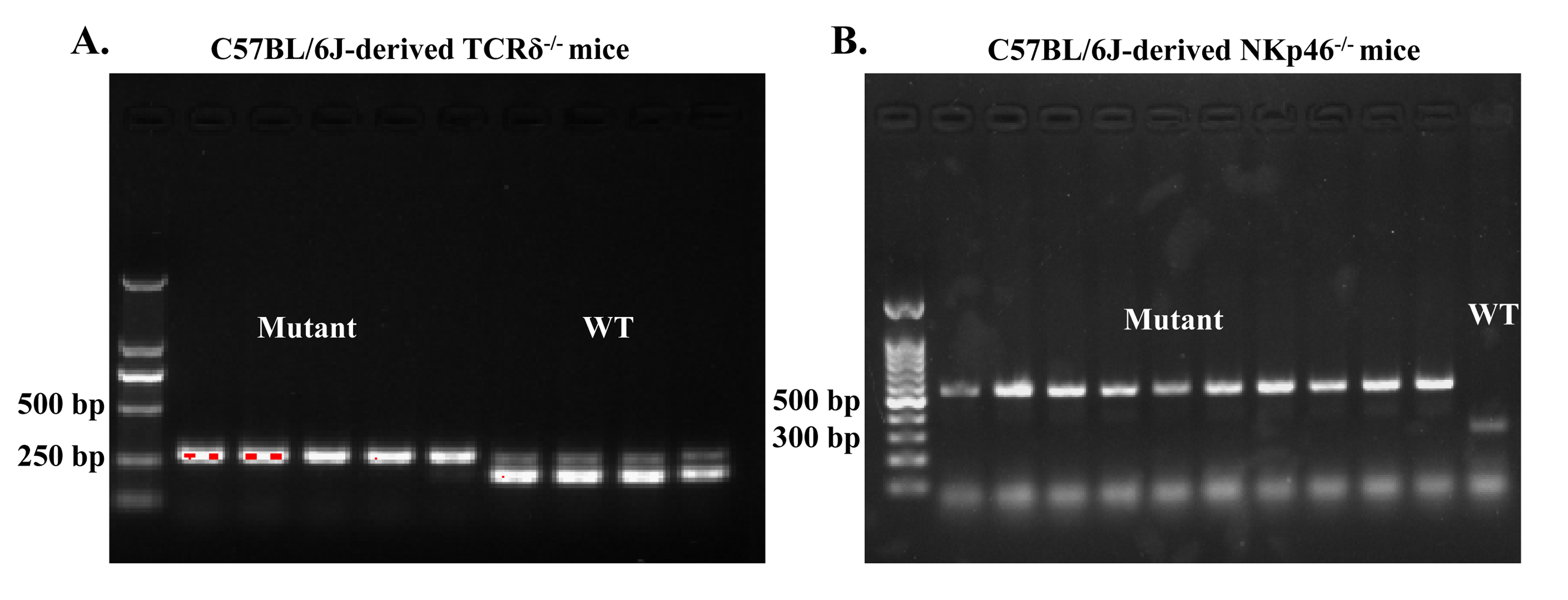
**

**Supporting Figure 1.** The genotype identification of TCRδ^-/-^ mice **(A)**, and NKp46^-/-^ mice **(B)** by gel electrophoresis on a 1.5% agarose gel.

**
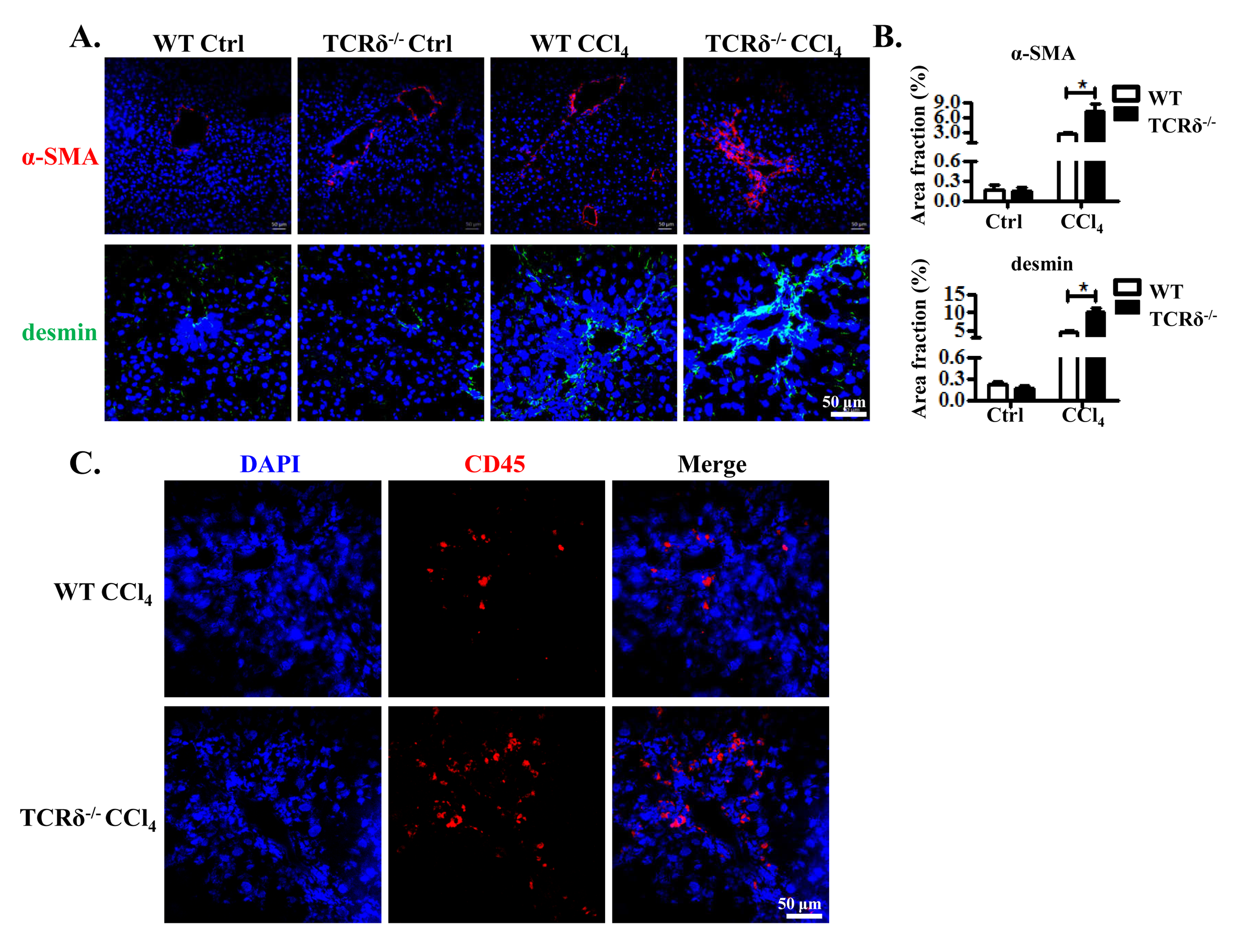
**

**Supporting Figure 2.** HSCs activation and leukocyte infiltration in WT and TCRδ^-/-^ mice upon chronic liver injury. **(A)** Representative immunofluorescence staining for α-SMA (red) or desmin (green) of liver frozen sections from WT and TCRδ^-/-^ mice treated with or without CCl_4_ for 4 weeks, and nuclei were counterstained with DAPI (blue). **(B)** Quantification of α-SMA and desmin-positive areas measured from five randomly nonoverlapping fields (n = 5). **P* < 0.05. * Compared with the control. (C) Representative immunofluorescence staining for CD45 (red) of liver frozen sections from CCl_4_-treated WT and TCRδ^-/-^ mice. Nuclei were counterstained with DAPI (blue).

**
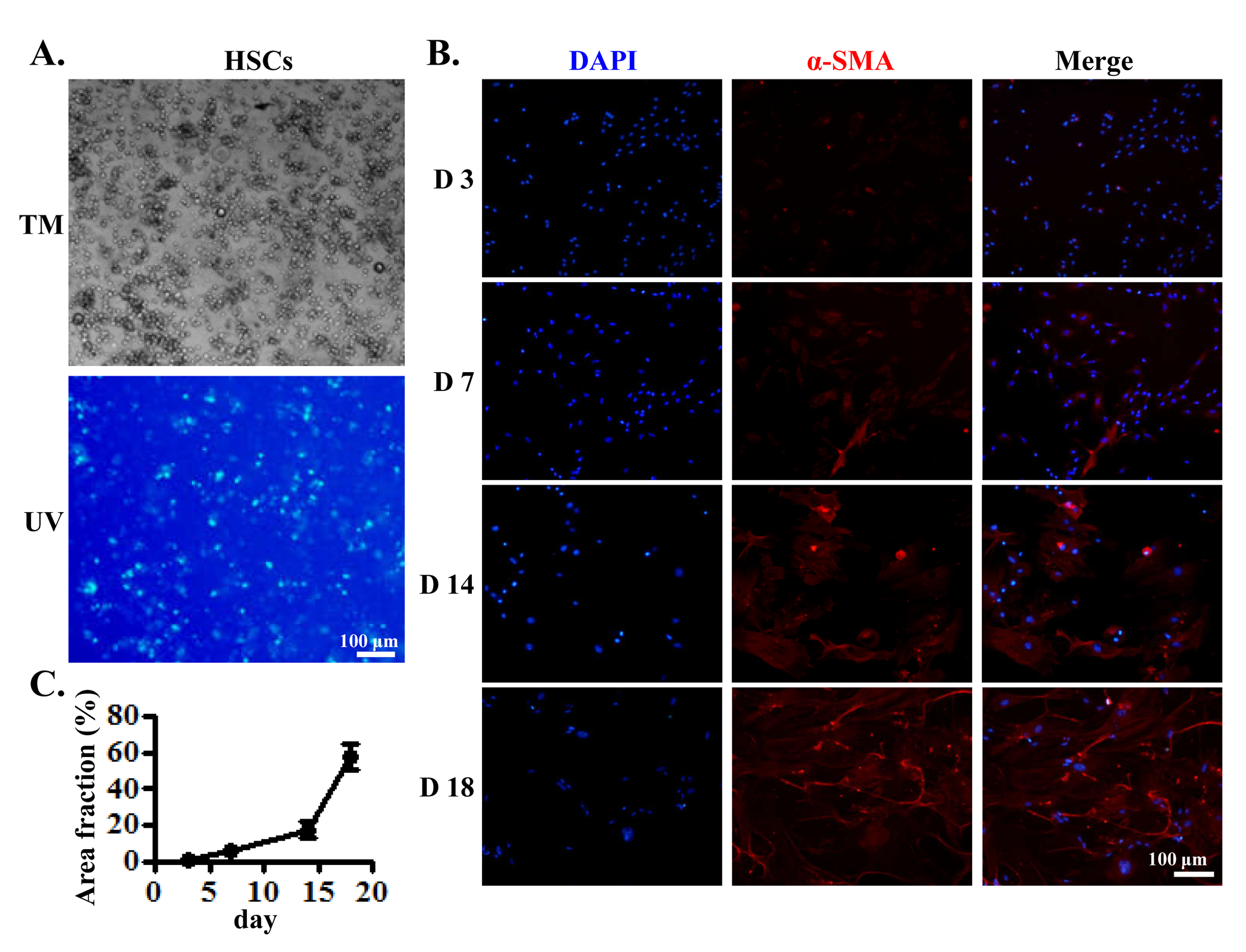
**

**Supporting Figure 3.** The characteristics of HSCs in culture. **(A)** The freshly isolated primary HSCs exhibit a positive signal (autofluorescence) in the UV-channel. TM: transmission light, UV: ultraviolet light. **(B)** The expression of α-SMA on isolated primary HSCs after cultivating for 3 (D3), 7 (D7), 14 (D14), and 18 (D18) days. **(C)** Quantification of the expression of α-SMA as shown in (B).

**
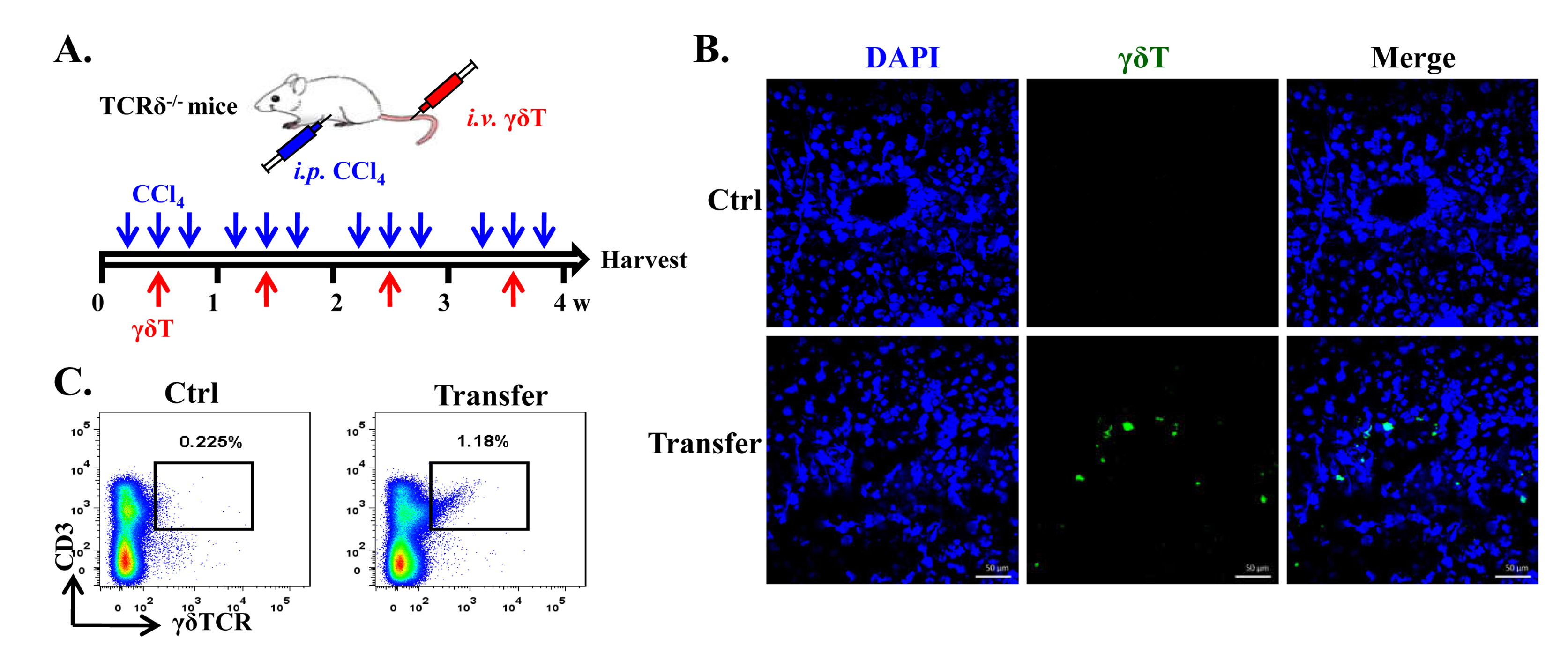
**

**Supporting Figure 4.** Adoptive transfer of hepatic γδT cells from WT mice into TCRδ^-/-^ mice during fibrosis. **(A)** Schematic diagram for adoptive transfer of γδT cells into TCRδ^-/-^ mice. Hepatic γδT cells were isolated from WT mice and intravenously injected into TCRδ^-/-^ mice once a week during CCl_4_ stimulation. **(B)** Immunofluorescence staining for γδT cells in frozen liver sections from TCRδ^-/-^ mice after transferring γδT cells (Transfer) or the same volume of normal saline (Ctrl) for 24 hours. **(C)** Representative FACS plots of γδT cells in livers of TCRδ^-/-^ mice after transferring γδT cells (Transfer) or normal saline (Ctrl) for 24 hours.

**
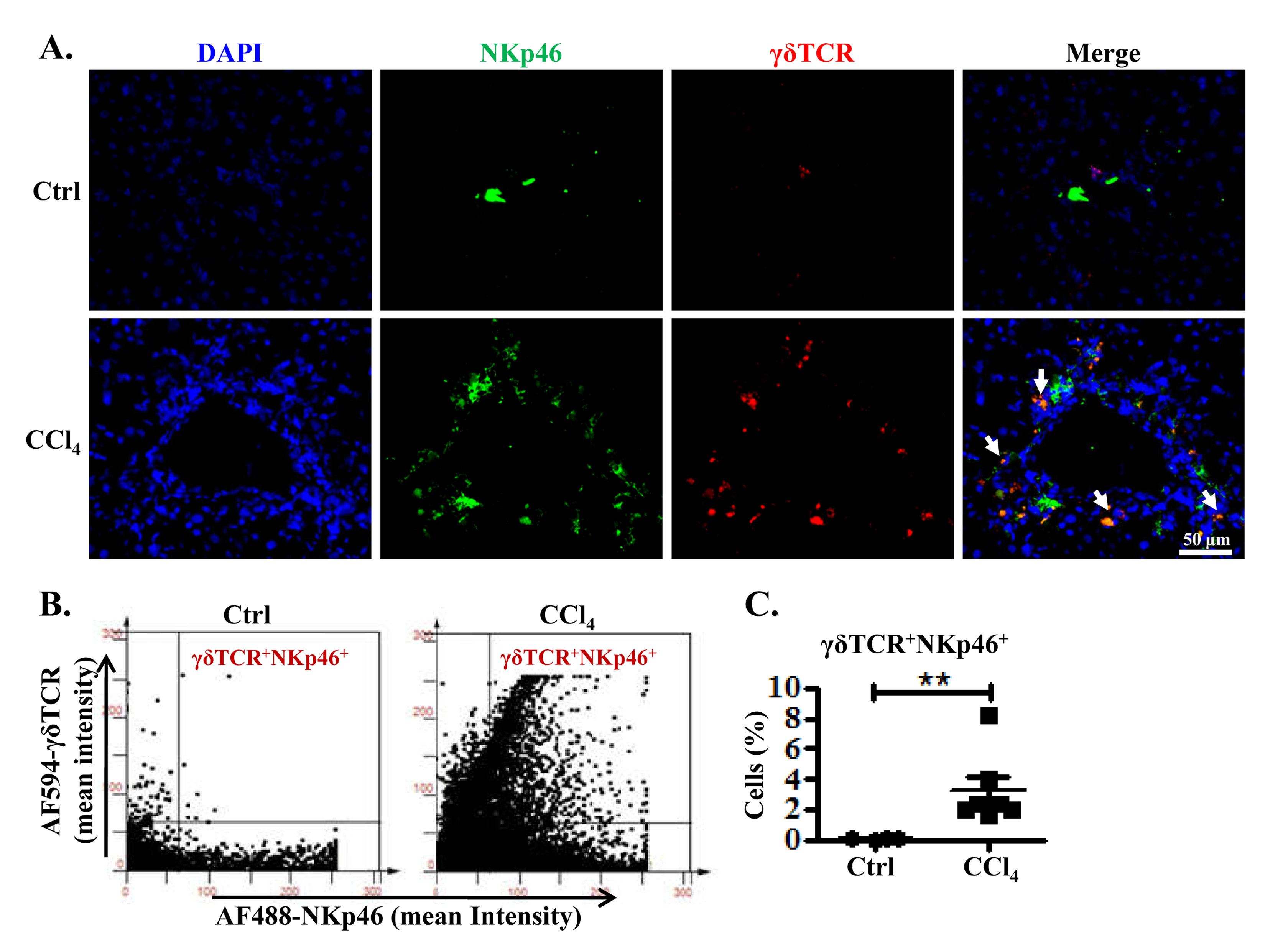
**

**Supporting Figure 5.** Hepatic γδT cells can acquire NKp46 expression following CCl_4_-induced liver fibrosis. **(A)** Representative images of fibrotic liver sections (n = 4) stained for γδTCR (red) and NKp46 (green) and counterstained with DAPI (blue). An arrow indicates representative γδTCR and NKp46 double positive cells. These images were scanned using the StrataFAXS Plus system and analyzed using StrataQuest. Scale bars, 50μm. **(B)** Scattergram of the analyzed immunofluorescence samples in (A), discrimination of NKp46^+^ γδT cells because of their mean intensity depicted in double positive region. **(C)** Statistical analysis of percentage of γδTCR and NKp46 double positive cells in total hepatic cells from fibrotic livers (CCl_4_) and normal livers (Ctrl). ***P* < 0.01.

**
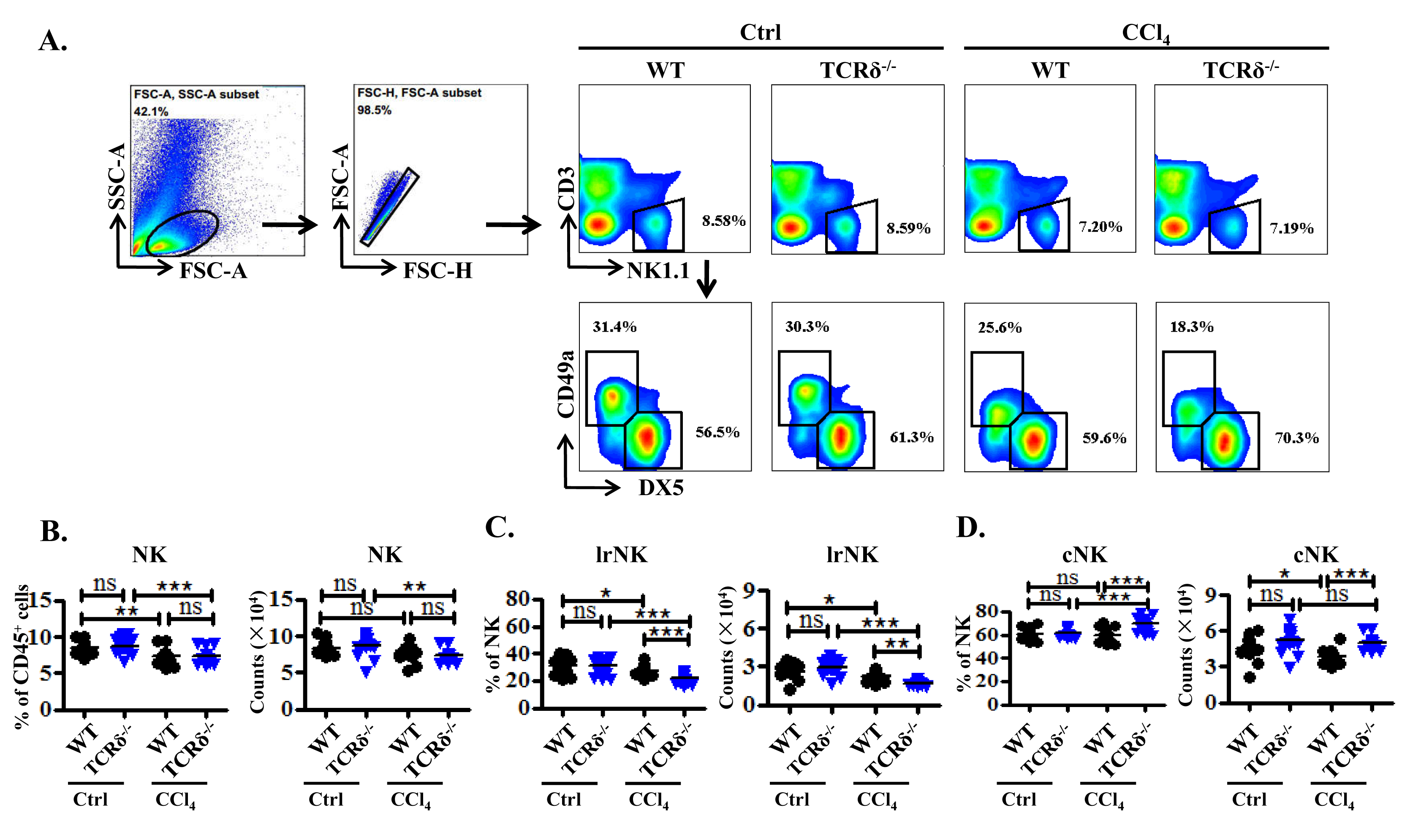
**

**Supporting Figure 6.** γδT cell deficiency alters the composition of NK cell subpopulations. **(A)** Representative FACS plots of CD3^-^NK1.1^+^ NK cells, CD3^-^NK1.1^+^CD49a^+^DX5^-^ lrNK cells and CD3^-^NK1.1^+^CD49a^-^DX5^+^ cNK cells in livers of corn oil- and CCl_4_-induced WT or TCRδ^‑/-^ mice. **(B)** Statistical analysis of the percentage and cell numbers of total NK cells. **(C)** Statistical analysis of percentage and cell numbers of lrNK cells. (D) Statistical analysis of percentage and cell numbers of cNK cells. Data are shown as the mean ± SEM. (n =15–18). **P* < 0.05, ***P* < 0.01, ****P* < 0.001.
